# Attentional bias towards and away from fearful faces is modulated by developmental amygdala damage

**DOI:** 10.1016/j.cortex.2016.04.012

**Published:** 2016-08

**Authors:** Morteza Pishnamazi, Abbas Tafakhori, Sogol Loloee, Amirhossein Modabbernia, Vajiheh Aghamollaii, Bahador Bahrami, Joel S. Winston

**Affiliations:** a Iranian Center of Neurological Research, Department of Neurology, Imam Khomeini Hospital, Tehran University of Medical Sciences, Tehran, Iran; b Students' Scientific Research Center, Tehran University of Medical Sciences, Tehran, Iran; c Department of Psychiatry, Icahn School of Medicine at Mount Sinai, New York, USA; d Roozbeh Psychiatric Hospital, Tehran University of Medical Sciences, Tehran, Iran; e UCL Institute of Cognitive Neuroscience, University College London, London, United Kingdom; f Wellcome Trust Centre for Imaging Neuroscience, University College London, London, United Kingdom

**Keywords:** Amygdala, Spatial attention, Urbach–Wiethe disease, Dot-probe, Emotional processing

## Abstract

The amygdala is believed to play a major role in orienting attention towards threat-related stimuli. However, behavioral studies on amygdala-damaged patients have given inconsistent results—variously reporting decreased, persisted, and increased attention towards threat. Here we aimed to characterize the impact of developmental amygdala damage on emotion perception and the nature and time-course of spatial attentional bias towards fearful faces. We investigated SF, a 14-year-old with selective bilateral amygdala damage due to Urbach–Wiethe disease (UWD), and ten healthy controls. Participants completed a fear sensitivity questionnaire, facial expression classification task, and dot-probe task with fearful or neutral faces for spatial cueing. Three cue durations were used to assess the time-course of attentional bias. SF expressed significantly lower fear sensitivity, and showed a selective impairment in classifying fearful facial expressions. Despite this impairment in fear recognition, very brief (100 msec) fearful cues could orient SF's spatial attention. In healthy controls, the attentional bias emerged later and persisted longer. SF's attentional bias was due solely to facilitated engagement to fear, while controls showed the typical phenomenon of difficulty in disengaging from fear. Our study is the first to demonstrate the separable effects of amygdala damage on engagement and disengagement of spatial attention. The findings indicate that multiple mechanisms contribute in biasing attention towards fear, which vary in their timing and dependence on amygdala integrity. It seems that the amygdala is not essential for rapid attention to emotion, but probably has a role in assessment of biological relevance.

## 1 Introduction

Evolutionary pressure ensures that in systems with limited perceptual capacity, stimuli that indicate potential environmental dangers receive privileged access to resources (Dolan, 2002, Öhman and Mineka, 2001). Numerous studies show that attention is preferentially oriented towards and maintained for longer by threat-related items (Yiend, 2010). Such attentional bias has been documented using a variety of stimuli (e.g., facial expressions, words, scenes) (Yiend, 2010) and evidence shows that threat-related stimuli affect both engagement and disengagement components of attention (Cisler et al., 2009, Koster et al., 2004a, Yiend, 2010). Attentional biases are observed at time-scales encompassing both automatic and strategic stages of information processing (Cisler and Koster, 2010, Cisler et al., 2009, Koster et al., 2005). Abnormal attention orienting to threat is a characteristic feature of anxiety disorders (Cisler and Koster, 2010, Salum et al., 2013, Shechner et al., 2012) and attentional bias modification has a role in anxiety treatment (Hakamata et al., 2010). However, the precise neural mechanisms that underlie attentional bias towards threat-related stimuli remain unclear.

The current literature on the neural mechanisms of attention to threat presumes a pivotal role for the amygdala (Pourtois, Schettino, & Vuilleumier, 2013). It is argued that the amygdala's bidirectional connections with sensory areas enhance perceptual processing of emotional stimuli (Freese and Amaral, 2009, LeDoux, 2007, Vuilleumier, 2005) and amygdala is therefore responsible for early (“automatic”) facilitated engagement of attention to threat (Cisler and Koster, 2010, Vuilleumier, 2005). Findings suggest that the later strategic stages of attention to threat and the disengagement component of attentional bias are controlled by higher-order cortical networks, predominantly the prefrontal attentional network (Cisler and Koster, 2010, Pourtois et al., 2013). Neuroimaging studies show that the enhanced cortical activations in response to fearful faces are absent in amygdala-damaged patients (Rotshtein et al., 2010, Vuilleumier et al., 2004) and support the role of amygdala in threat-related attention. However the causal involvement of amygdala in biasing attention to emotion has not been confirmed (Pessoa & Adolphs, 2010). The handful of behavioral experiments on amygdala-damaged patients have given inconsistent results. Out of seven published studies (Anderson and Phelps, 2001, Bach et al., 2015, Bach et al., 2011, Piech et al., 2010, Piech et al., 2011, Terburg et al., 2012, Tsuchiya et al., 2009), only two provide positive evidence for impaired attention to threat after amygdala damage (Anderson and Phelps, 2001, Bach et al., 2015). In an early influential study, Anderson and Phelps (2001) showed that a patient with non-selective bilateral temporal lobe lesions did not exhibit facilitated attention to aversive words during the attentional blink task. However, testing the same task on two patients with focal amygdala lesions failed to replicate this effect (Bach et al., 2011). Two other experiments, one using attentional blink with pictures (Piech et al., 2011) and the other using continuous flash suppression paradigm (Tsuchiya et al., 2009, experiment 3) also report that threat-related attentional bias persists despite amygdala damage. Another piece of positive evidence comes from a visual search paradigm that showed impaired attention to angry faces after amygdala damage (Bach et al., 2015). However, two other studies that employed visual search with fear-related targets did not find any deficit in similar patients (Piech et al., 2010, Tsuchiya et al., 2009, experiment 2). Adding to the disparity within the literature, there is one report of increased attention to fear in five patients with lesions relatively selective to basolateral amygdala (Terburg et al., 2012). These inconsistencies warrant further investigations to explain the exact role of amygdala in triggering and maintaining the attentional bias towards threat. Particularly, what is lacking is a clear characterization of behavioral consequences of amygdala damage based upon the components of attentional bias and the stages of information processing (Cisler and Koster, 2010, Pourtois et al., 2013).

In the current study, we aim to characterize emotion perception and the temporal dynamics of spatial orienting towards fearful faces in an adolescent patient with selective bilateral amygdala damage due to Urbach–Wiethe disease (UWD) compared to a *N* = 10 control group. UWD is a rare genetic condition that causes focal symmetrical calcifications in amygdala bilaterally with sparing of other brain regions (Appenzeller et al., 2006). Several previous cases of children and adolescents with bilateral amygdala damage have been reported (Emsley and Paster, 1985, Ito et al., 2000, Omrani et al., 2012, Savage et al., 1988). However, very little information could be found on the cognitive consequences of amygdala damage at young ages. In particular, the attentional bias to threat has been solely investigated in adult amygdala-damaged patients and few neuropsychological assessments of adolescent patients have mainly focused on deficits in emotion recognition and memory (Steenberg, 2014, Thornton et al., 2008). Attentional bias to threat begins very early in life (Creswell et al., 2008, LoBue and DeLoache, 2010) and is consistently observed across age groups (preschoolers: LoBue, 2009; preteens: Waters, Lipp, & Spence, 2004; and adolescents: Wolters et al., 2012). Threat bias appears to be present in early childhood as a core function that facilitates survival and adaptive social behavior (LoBue & Rakison, 2013), but biases then change as a function of development (Field & Lester, 2010). With increasing age, moderating factors such as trait anxiety, past experiences and environmental events seem to have a larger effect on the strength and direction of attentional biases (Field and Lester, 2010, Shechner et al., 2012). However, the neural mechanisms underlying attention to threat seem not to change during development (Lindstrom et al., 2009).

We first explored the emotional experience of our patient using a fear sensitivity questionnaire and a facial expression classification task. Next, to test the spatial orientation of attention, we adopted the ‘dot-probe’ double cuing task (MacLeod, Mathews, & Tata, 1986). This task allows drawing inferences about the engagement and disengagement of attention (Koster, Crombez, Verschuere, & De Houwer, 2004) and can illuminate both automatic and strategic stages of attentional bias by employing short and long cue exposure durations (Koster et al., 2005). In the dot-probe task, targets are presented either at the same or opposite to the location of a preceding emotionally salient cue. The difference in reaction time (RT) to targets located at congruent versus incongruent location relative to the cue is interpreted as the bias of spatial attention (i.e., ‘vigilance’ or ‘avoidance’). We employed the dot-probe task with face-pair cues that could both be neutral (baseline) or comprise a neutral and fearful face. We used three cue exposure durations (100, 500, 1000 msec) to examine the time-course of attentional bias. Assuming that the amygdala's contribution in directing attention is more critical at early stages of information processing, we expected to find disparate impacts of amygdala damage on attentional bias at short versus late time-points.

## 2 Materials and methods

### 2.1 Participants

**Fig. 1:**
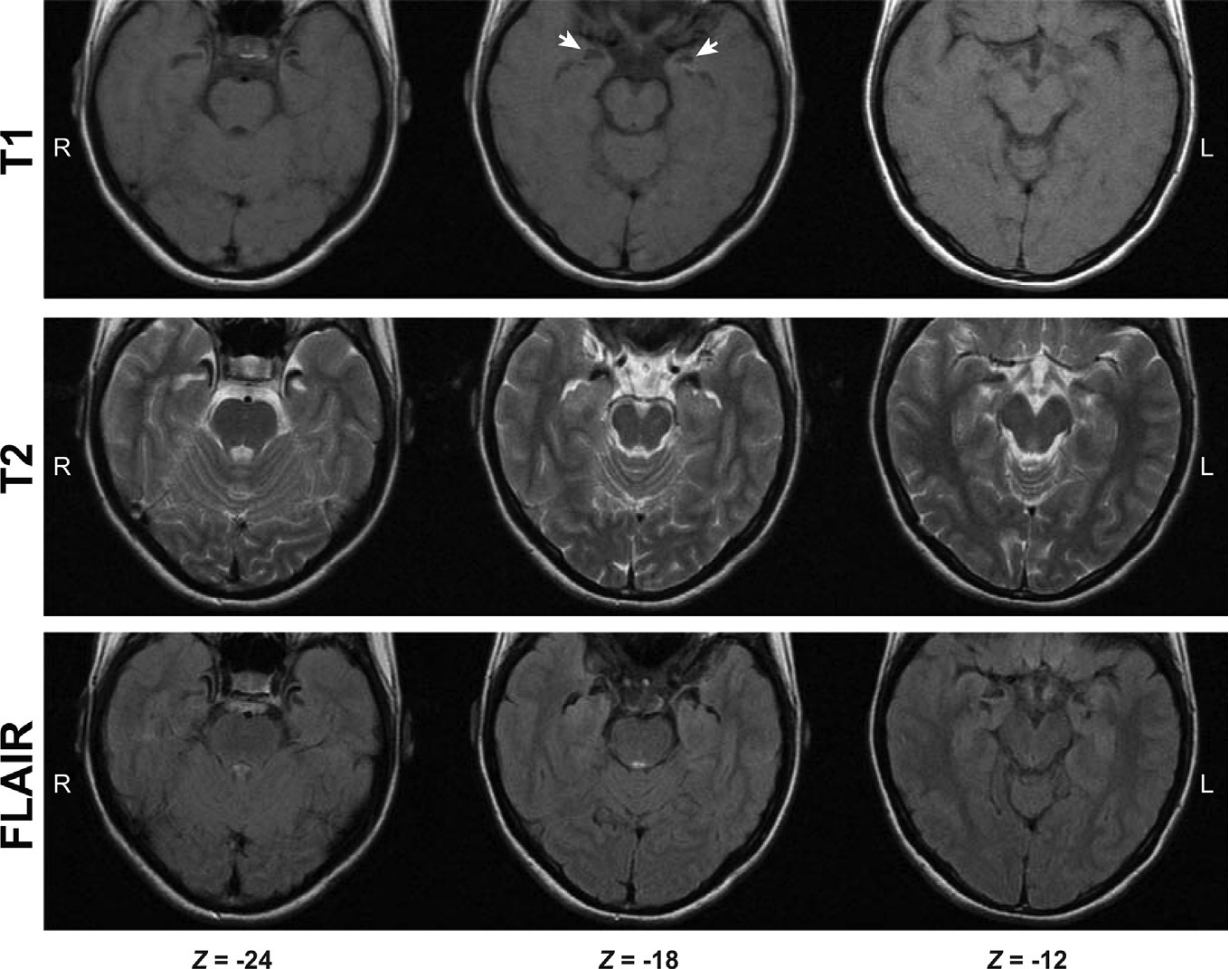
**T1, T2, and FLAIR sequence MRI of SF**. Images demonstrate bilateral amygdala lesions (arrowheads) as a result of symmetrical calcifications due to Urbach–Wiethe disease. Each column presents corresponding axial sections; from left to right at 24, 18, and 12 millimeters below the anterior commissure. Images are in radiological convention.

Patient SF (female, 14.5 years old at the time of testing) was diagnosed with UWD after investigations for epilepsy showed bilateral amygdala lesions (Omrani et al., 2012) (Fig. 1). She had a 10-year history of focal seizures but had been drug- and symptom-free for 8 months when tested. Psychiatric evaluation of SF did not converge to any diagnosis but revealed histories of two interpersonal traumatic events, three and four years ago, and a history of suicidal ideations, with a plan as recently as a month prior to the study (for more details see Supplementary Material §1). Ten female participants, matched for gender (female), handedness (right handed), age (M ± SD = 14.8 ± .2), education (8.5 years of formal schooling), home language (Persian), and socioeconomic level were recruited as control subjects. A physician interviewed the control group to confirm psychiatric and neurologic health. To measure everyday fear sensitivity, SF and control participants completed the Fear Survey Schedule for Children–Revised (FSSC-R) (Ollendick, 1983). The Ethics Committee at the Tehran University of Medical Sciences approved all procedures and informed consent was obtained from all participants.

### 2.2 Facial expression classification

Color face images from Radboud Face Database (RaFD) (Langner et al., 2010) were employed. A set of 234 images [39 Identities (19 females) × 6 Expressions: ‘happy’/‘sad’/‘fearful’/‘angry’/‘surprised’/‘disgusted’] were presented in random order. On each trial one image was displayed on a black background with all the adjectives (in Persian) displayed alongside on the right. Participants selected the best-fitting label by mouse, with no time limit.

### 2.3 Emotional dot-probe task

A subset of RaFD images (27 models; 12 female; fearful and neutral expressions) were used. Faces were grayscale-transformed, equalized for intensity and contrast, and cropped to eliminate hair and other features falling outside the oval borders (6° main diagonal).

**Fig. 2:**
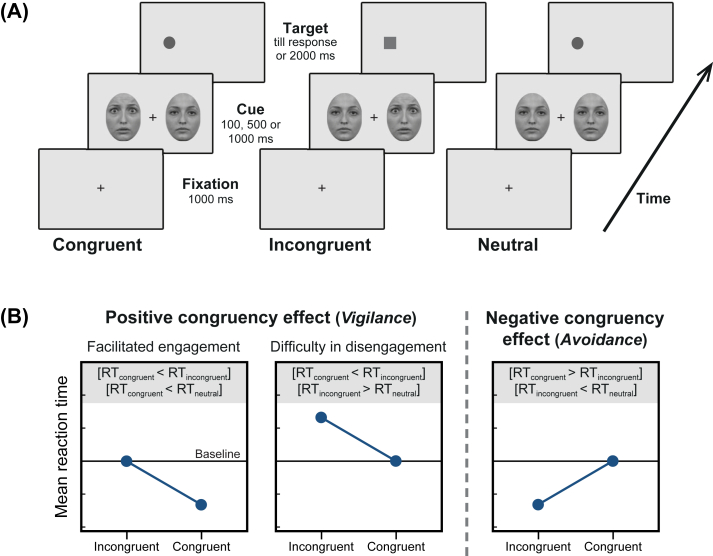
**Stimuli sequence, experimental conditions, and alternative results of the dot-probe task**. **(A)** Each trial started with a fixation cross. Each cue display consisted of a pair of face image of the same identity. On neutral trials (right), the same face with a neutral expression was displayed on both sides. In the other two conditions, one of the two faces was fearful. In congruent trials (left), the target appeared on the same side as the fearful face. In incongruent trials (middle) the target appeared on the opposite hemifield. Three cue durations (100, 500, and 1000 msec) occurred with equal probability. Stimuli are not drawn to scale. **(B)** Schematics of typical behavioral results obtained in dot-probe tasks. Significant difference between reaction times (RT) in congruent and incongruent trials indicates an attentional bias towards (left and middle panels) or away (right panel) from fear. A positive congruency effect could be due to either ‘facilitated engagement’ (left) or ‘difficulty in disengagement’ (middle). Comparison with baseline (RT in neutral trials; horizontal black lines) is necessary to determine the affected components of attentional bias.

Each trial (Fig. 2A) started with a central black fixation cross (.2° × .2°, 5 cd/m^2^; duration 1000 msec) on a uniform gray background (15 cd/m^2^). Subsequently, two face stimuli (same identity) were presented at 7° eccentricity to the left and right of fixation. To probe the time-course of attentional effects, three cue durations (100, 500, or 1000 msec) were used. On disappearing, the cue was replaced immediately by the target stimulus. The target was a circle or square (.5° × .5°; dark-gray, 10 cd/m^2^) that appeared in the left or right visual field (LVF, RVF) at 7° eccentricity with equal probability, and participants were instructed to maintain central fixation and report the target's shape by pressing the designated keyboard buttons. Accuracy and speed were equally emphasized.

We tested three conditions: ‘congruent’, ‘incongruent’, and ‘neutral’. On neutral trials, the same face with a neutral expression was displayed on both sides. In the other two conditions, one of the two faces was fearful. In congruent trials (Fig. 2A; left), the target appeared on the same side as the fearful face. In incongruent trials (Fig. 2A; middle) the target appeared on the opposite hemifield. In total each participant completed 1440 trials over two testing sessions, each lasting approximately 40 min. Each configuration (Cue duration × Trial type) occurred with equal probability in random order.

Based on previous studies (Mogg & Bradley, 1998), we reasoned that a positive congruency effect [RT_congruent_ < RT_incongruent_] would indicate ‘vigilance’ to fear whereas the reverse effect would indicate fear ‘avoidance’. Comparison with a baseline condition (without emotional cueing) is necessary to determine the components of attentional bias (i.e., ‘engagement’ or ‘disengagement’) (Koster, Crombez, Verschuere, et al., 2004). A positive congruency effect could be either due to ‘facilitated engagement’ [RT_congruent_ < RT_neutral_] (Fig. 2B; left) or ‘difficulty in disengagement’ [RT_incongruent_ > RT_neutral_] (Fig. 2B; middle).

### 2.4 Statistical considerations

Analysis of single-case experiments requires special statistical methods (McIntosh & Brooks, 2011). We employed the modified *t*-test proposed by Crawford and Howell (1998) to test the significance of the deficits in SF's fear sensitivity score and expression classification performance. This procedure is particularly suited for comparing a single observation with the mean of a small control group (Crawford and Garthwaite, 2006, Crawford and Garthwaite, 2012). The logic behind Crawford & Howell's method can be extended to analysis of variance (ANOVA) procedure (Corballis, 2009b), and is valid for factorial analysis of scores measured under several conditions of the *same* task (Corballis, 2009a, Crawford et al., 2009). See Supplementary Material (§5) for further details and discussion of alternative statistical methods. We applied ANOVA on mean reaction times of subjects to test for main effects and interactions between conditions of the dot-probe experiment. For pairwise comparison between mean reaction times of SF in each trial type (congruent, incongruent, neutral) we used the Crawford and Garthwaite's revised test for difference (Crawford & Garthwaite, 2005). This method is a modified paired-sample *t*-test suited for comparing a patient's performance on parallel versions of a task with that of controls under two different experimental conditions. Corresponding pairwise comparisons for control subjects were run using conventional paired *t*-tests. For confirmation, we reanalyzed SF's dot-probe data using trial-by-trial reaction times (i.e., not averaged over conditions) and conventional statistical methods (Supplementary Material §6). IBM SPSS Statistics (Ver. 20.0) was used for data analysis. In SPSS software, the Crawford and colleagues methods are applied by defining the single case as a group of *N* = 1 and no further adjustment is required (Corballis, 2009a). In all tests *p*-values < .05 were considered significant (with Bonferroni adjustment where appropriate).

## 3 Results

### 3.1 Fear sensitivity

**Fig. 3:**
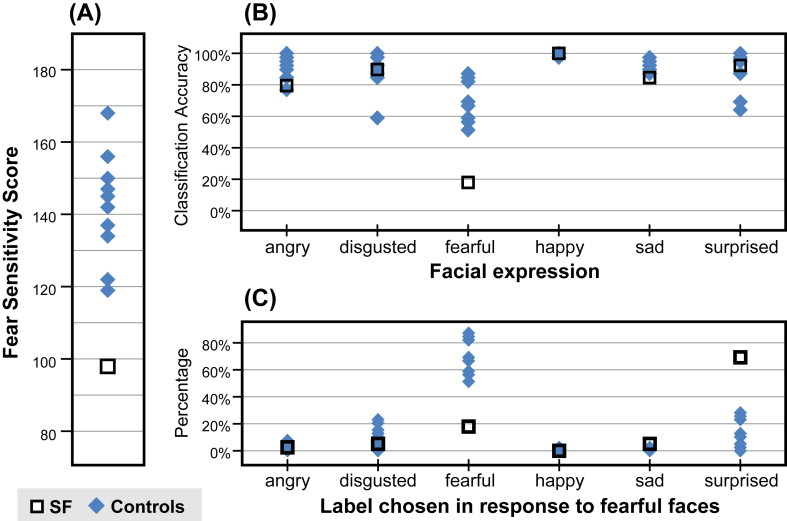
**Deficits in experiential emotion processing with developmental amygdala damage**. **(A)** Participants' scores from the Fear Survey Schedule for Children–Revised (FSSC-R). SF had a significantly lower score, suggesting that she had lower everyday experience of fear. **(B)** Performance accuracy in the facial expression classification task. SF was specifically impaired in classifying the fearful facial expression. **(C)** Distribution of labels assigned to fearful faces. SF assigned the ‘surprised’ label to 69% of the fearful faces.

The FSSC-R questionnaire lists 80 specific situations or objects (e.g., “getting lost in a strange place”, “snakes”, etc.). Participants described how much they fear each item (“none”/“some”/“a lot”; scored 1–3 respectively). SF scored 98, reporting “a lot” of fear for only three items (see Supplementary Material §1), while controls' scored significantly higher [M ± SD = 142 ± 14.8; range: 119–168; *t*(9) = 2.80; *p* = .02] (Fig. 3A).

### 3.2 Facial expression classification

With the exception of fearful expressions, SF and controls were equally accurate (all *p* > .05) in identifying the relevant emotional label for the faces (Fig. 3B). When a fearful face was presented, SF chose the correct label in only 18% of trials, significantly lower than the average performance of controls [72%; *t*(9) = 3.85; *p* = .004]. SF categorized fearful faces as ‘surprised’ in 69% of trials; whereas controls had a broader distribution of errors (Fig. 3C).

### 3.3 Emotional dot-probe task

Errors in reporting the shape of the target were rare. On average, controls made an error on .9% of trials (SD = .8). SF had a significantly higher error rate [3.1%; *t*(9) = 2.53; *p* = .032]. Prior to averaging RTs, error trials and trials with outlier RTs were excluded. Outliers were defined separately for each participant as RTs that deviated more than 1.5 inter-quartile ranges from the upper and lower quartiles. These trials comprised 4.2% of SF's data and 2.5% of all collected data. We found no evidence for speed-accuracy trade-off (see Supplementary Material §4).

**Fig. 4:**
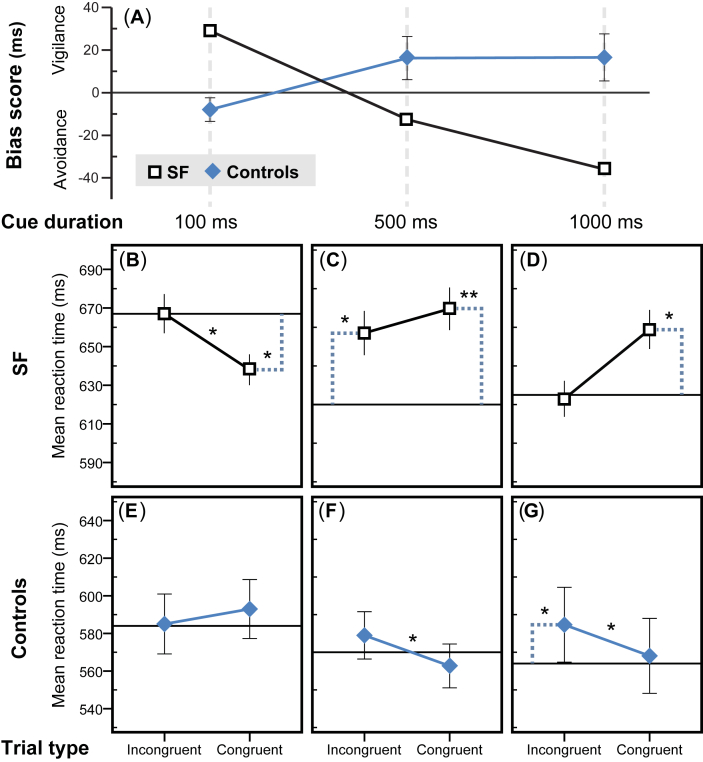
**Results of the dot-probe task**. Panel **(A)** shows the attentional bias scores of SF and controls at each cue exposure duration. Bias score is calculated by subtracting reaction times on congruent trials from reaction times on incongruent trials. Positive attentional bias scores indicate attention towards the fearful face. Negative scores indicate avoidance of fear location. Panels **(B–G)** show mean reaction time of SF and controls on congruent, incongruent, and neutral trials at each cue exposure duration. SF showed attentional bias towards fearful faces mediated by *facilitated engagement* effect at 100 msec **(B)**; generally slower reaction times in trials including a fearful face, but no significant attentional bias at 500 msec **(C)**; and bias to avoid the location of fearful faces, probably due to *inhibition of return* at the exposure duration of 1000 msec **(D)**. Controls showed a trend to avoid fearful faces at 100 msec **(E)**; significant attentional bias was observed afterwards at 500 msec **(F)**; and at 1000 msec mediated by *difficulty to disengage* from the location of fearful faces **(G)**. Horizontal black lines indicate the mean reaction time at neutral trials. Dotted lines represent significant difference with baseline. Error bars show ± standard error of mean, representing within-subject variance in SF (uncapped bars) and between-subject variance in controls (capped bars). *p < .05, **p < .01.

Mean RTs for each experimental condition (Supplementary Table 1) were entered into a 3-way repeated measures ANOVA with Group (controls/SF) × Cue duration (100/500/1000 msec) × Congruency (congruent/incongruent) as factors. None of the main effects nor the 2-way interactions were significant. However, a significant 3-way interaction [*F*(2, 18) = 9.77; *p* = .001] showed that the temporal pattern of emotion–attention interaction differed between SF and controls. In follow-up tests, the Cue duration × Congruency interaction was examined within SF and the control group separately and showed temporal mediation of attentional effects in both SF [*F*(2, 18) = 5.42; *p* = .014] and controls [*F*(2, 18) = 9.89; *p* = .001]. Note that in this analysis the RT from neutral trials are not included as they cannot be differentiated as being congruent or incongruent. Attentional bias scores [RT_congruent_−RT_incongruent_] for SF and controls at each cue duration are presented in Fig. 4A. As mentioned earlier, comparison with neutral trials' RT (i.e., baseline RT unaffected by attentional cueing) is necessary to determine which component of spatial attention is affected (Koster, Crombez, Verschuere, et al., 2004). To reveal the attentional behavior of SF and controls at each cue duration, we performed pairwise comparisons between all the three trial types. Including the baseline condition tripled the number of planned tests. We used Bonferroni adjustment to control the probability of false positives.

#### 3.3.1 SF

With the shortest cue duration (100 msec), SF showed a positive congruency effect [*t*(9) = 3.31; *p* = .027] implying rapid vigilance for fear. This attentional bias disappeared with longer cue durations [500 msec: *t*(9) = .78; *p* > .1; 1000 msec: *t*(9) = 2.06; *p* > .1]. Pairwise comparison with baseline confirmed that at cue durations of 100 msec, SF showed facilitated engagement to fear location (RT_congruent_ < RT_neutral_ [*t*(9) = 2.96; *p* = .048]; no significant difference between RT_incongruent_ and RT_neutral_ [*t*(9) = 0]) (Fig. 4B). When cue duration was 500 msec, there was no bias but compared to the neutral condition, SF responded more slowly in the emotional trials with significantly longer RTs in both congruent [*t*(9) = 4.51; *p* = .004] and incongruent [*t*(9) = 3.16; *p* = .035] trials (Fig. 4C). With the longest cue duration (1000 msec), the congruency effect was not statistically significant. Comparison with the neutral condition showed a significant delay in responding to congruent trials [*t*(9) = 2.96; *p* = .048] but not incongruent trials [*t*(9) = .11] (Fig. 4D).

#### 3.3.2 Controls

With the shortest cue duration (100 msec), controls showed a marginal effect of fear avoidance [*t*(9) = 2.87; *p* = .056]. Longer cue durations resulted in significant attentional bias towards fear at both 500 msec [*t*(9) = 3.20; *p* = .032] and 1000 msec [*t*(9) = 3.00; *p* = .045] conditions. Comparison with baseline revealed a trend for longer RTs in *congruent* trials in the 100 msec condition [*t*(9) = 2.79; *p* = .064] (Fig. 4E). At cue durations of 500 msec there was no significant difference between either congruent or incongruent conditions and the baseline (Fig. 4F). At cue durations of 1000 msec the mean RT in incongruent trials was significantly longer than neutral baseline [*t*(9) = 3.77; *p* = .013] suggesting that controls had difficulty in disengaging fear location (Fig. 4G).

## 4 Discussion

We investigated SF, a 14-year-old female with bilateral amygdala lesions due to UWD, and ten matched controls. Psychiatric evaluation of SF revealed no pathological diagnosis. The fear survey revealed her significantly lower fear sensitivity. These findings are consistent with prior reports from an adult UWD patient (Feinstein et al., 2011, Tranel et al., 2006). Moreover we found that SF is specifically impaired in classifying the fearful facial expressions, a frequent finding after the damage of amygdala either due to UWD (Adolphs et al., 1994, Becker et al., 2012, Siebert et al., 2003) or other less selective pathologies (Schmolck and Squire, 2001, Sprengelmeyer et al., 1999).

Our main aim was to investigate the causal contribution of amygdala to the orienting of spatial attention by fearful faces. We measured attentional bias using a dot-probe task with congruent and incongruent cues and used various cue durations to investigate the temporal dynamics of attentional biases. To discriminate between engagement and disengagement components of attention, we included trials with neutral/neutral face pairs to measure baseline RTs. The results revealed that SF and controls demonstrated opposite patterns of attentional biases in the early and late time-points after attentional cue onset (Fig. 4A). SF showed attentional bias towards fear at the shortest tested cue duration of 100 msec. In controls, the attentional bias towards fearful faces was observed in the middle and longer time windows (500–1000 msec post-cue). In contrast, SF showed no bias at the moderate cue durations, and only a weak bias away from the fearful cue location at 1000 msec. These findings suggest that SF's attention was rapidly engaged to the fearful face but shortly afterwards her attention disengaged from the fear location (by a timescale of <500 msec post-cue) and proceeded to avoid the previously attended location possibly via a mechanism similar to ‘*inhibition of return*’ (Klein, 2000). Healthy subjects, on the other hand, showed difficulty in disengaging attention from the location of fearful faces (Fig. 4G). Our results reveal for the first time the separable effects of amygdala damage on engagement and disengagement components of spatial attention.

We found that the attentional bias in normal subjects was due to difficulty in disengaging attention from the location of fear. SF showed an early bias towards fear due to facilitated engagement of attention, but unlike the control group did not show disengagement cost at any of three measured time-points. This is a peculiar finding because abundant dot-probe data demonstrate that unlike disengagement effects, that might occur independently, facilitated engagement to emotion does not occur alone and is almost always followed by difficulty in disengagement (Cisler & Koster, 2010). Our results thus imply that amygdala damage abolishes the difficulty in disengaging from fear location at moderate to late time points, suggesting that amygdala function is necessary for the disengagement costs to occur. Electrophysiological and neuroimaging studies have begun to fractionate the neural underpinnings of the facilitated capture of spatial attentional by fearful faces and the attentional disengagement costs imposed by such stimuli, and are consistent with the suggestion that these effects have dissociable neural correlates (Pourtois et al., 2006, Pourtois et al., 2005). Future studies, could test whether these neural mechanisms are causally dependent upon amygdala projections. Our current results imply that amygdala actively increases the attentional dwell time on biologically significant signals.

Strikingly, we found rapid engagement of attention by fear in SF at the shortest cue duration. This attentional bias suggests that despite bilateral amygdala damage and impairment of fear recognition, fearful faces could nonetheless rapidly orient SF's spatial attention. Attentional orienting by such short cue durations suggests that a reflexive, bottom-up mechanism is still functional in SF. This is consistent with previous reports that the amygdala is not essential for rapidly detecting and attending to emotional stimuli (Bach et al., 2011, Piech et al., 2010, Piech et al., 2011, Tsuchiya et al., 2009). But assuming no role for the amygdala in orienting attention to emotion is problematic for interpreting multiple studies that showed that projections from the amygdala modulated perceptual and attentional responses to fear-related stimuli (Benuzzi et al., 2004, Rotshtein et al., 2010, Vuilleumier et al., 2004). Damage to amygdala abolishes fear-induced enhancement of early visual responses (Rotshtein et al., 2010) and these early enhancements appear functionally relevant to the attentional bias towards fear in the dot-probe paradigm (Pourtois, Grandjean, Sander, & Vuilleumier, 2004). So what is the function of amygdala-mediated enhancement of visual responses, if amygdala is not necessary for initial attention to fear?

Current theories propose that the function of amygdala is not specific to emotional processing, instead playing a role in optimizing the allocation of perceptual resources to stimuli based on biological value and goal relevance (Pessoa and Adolphs, 2010, Sander et al., 2003, Adolphs, 2008). From this perspective it is reasonable to think that amygdala might act to either facilitate or prevent orienting towards threat signals, by weighing up the cost of ignoring potential danger against the benefit of goal-directed tasks (Pessoa, 2009). Indeed we found that compared to SF, the shift of attention towards fear arose later in healthy controls. A brief task-irrelevant fearful face is a relatively weak signal of environmental danger—and is safe to ignore, as controls did in the 100 msec condition of our experiment. However, as the fearful face persists its biological significance increases; at longer cue durations it is sensible to interrupt the task and attend to the fearful face location—and engage with it until the potential source of threat is resolved. The delay shown by healthy control participants in orienting to fear fits this ecological perspective on amygdala function (Pessoa, 2009) and suggests that amygdala can actively act to suppress the fear bias when threat is weak and irrelevant. This claim is supported by at least one other study of several UWD patients which provided causal evidence that the basolateral amygdala nucleus is necessary to inhibit the reflexive distraction of attention by task-irrelevant threat signals (Terburg et al., 2012). Without a functional amygdala, SF showed reflexive attention to brief signals of fear and avoided the long-lasting signals of potential threat. These are both harmful strategies and suggest that she was impaired in adjusting attentional selection based on the biological significance of sensory events. The current set of findings corroborate the notion that amygdala is crucial for top-down guidance of spatial attention to biologically relevant and not necessarily emotional features of the visual scene (Jacobs et al., 2012, Pourtois et al., 2013). Remarkably, the failure in top-down guidance of attention seems to be the basis of impaired recognition of fearful faces, which is the hallmark deficit of amygdala-damaged patients. Studies on SM, the single-most studied UWD case (2008; 2005) suggest that amygdala-damaged patients are not impaired in perception of fear *per se*, but fail to properly attend to parts of face images that are relevant for correct expression recognition (Kennedy & Adolphs, 2011). Intriguingly, SM's fear recognition deficit was corrected after an explicit instruction to attend to the eye region of faces (Adolphs et al., 2005). Our patient mostly labeled ‘fearful’ faces as ‘surprised’ (see Fig. 3C). Compared to other expressions, there is more overlap between the facial features that relay fear and surprise emotions (Smith, Cottrell, Gosselin, & Schyns, 2005) and discriminating the two relies on active attentional selection (Schyns, Petro, & Smith, 2009). Therefore, SF's deficit in the facial expression classification task might also be consistent with a role for amygdala in top-down attentional guidance.

Here we discussed findings from a single case study. For this reason, caution should be exercised in interpreting the results for amygdala's attentional function based on this study alone. Further studies on patients with bilateral amygdala damage are needed to confirm current results. Several points should be noted in conducting future studies. First, the amygdala is a heterogeneous structure and animal studies have found disparate behavioral outcomes after lesions of specific subnuclei (Swanson & Petrovich, 1998). Precise characterization of location and extent of patient's lesions, might help reconcile the reports of diminished (Anderson & Phelps, 2001), preserved (Bach et al., 2011), and even increased attention to fear after amygdala damage (Terburg et al., 2012). Second, here we only used task-irrelevant fearful faces to cue attention. The consequences of amygdala damage on attentional orientation by task-relevant and more potent danger signals remain to be investigated. Third, we relied on changes in RT to study attentional effects. However, threat-signals affect both the latency (Pessoa, Padmala, Kenzer, & Bauer, 2012) and accuracy (Phelps, Ling, & Carrasco, 2006) of perceptual responses—occasionally in opposing directions (Bocanegra, 2014). The separable roles of amygdala in mechanisms underlying speed-accuracy trade-offs is an important, yet unstudied topic. Future research should focus on explicit characterization of the time-course, attentional components, and neural pathways that comprise interactions between amygdala and attentional effects (Cisler & Koster, 2010). This seems a promising approach for unraveling amygdala's functions, and it's role in pathophysiology of anxiety disorders (Birn et al., 2014, Milham et al., 2005). Abnormal attentional bias towards threat robustly relates to elevated trait anxiety (Bar-Haim et al., 2007, Hakamata et al., 2010), and future theories must address such relationships.

## 5 Conclusion

We showed that an adolescent patient with bilateral amygdala damage rapidly attended fearful faces, but disengaged from them prematurely. To our knowledge, this is the first demonstration of the separable effects of amygdala damage on engagement and disengagement components of spatial attention. Our findings show that attentional behavior is shaped by multiple influences from amygdala, occurring at distinct time points; and suggest that the amygdala has a modulatory role in threat-related attentional bias. It seems that the amygdala is not essential for rapid attention to emotion. Instead, the amygdala probably has a crucial role in assessing the biological relevance of sensory events, and is essential for efficient allocation of perceptual resources.

## Financial disclosure

The authors report no biomedical financial interests or potential conflicts of interest.
