# Supplementary material for: Attentional bias towards and away from fearful faces is modulated by developmental amygdala damage
Source: Cortex. 2016 Aug;81:24–34. doi: 10.1016/j.cortex.2016.04.012 (PMC4962776; doi:10.1016/j.cortex.2016.04.012)
Supplement: Supplementary file 1 [file mmc1.docx]

**Attentional bias towards and away from fearful faces is modulated by developmental amygdala damage**

Morteza Pishnamazi, Abbas Tafakhori, Sogol Loloee, Amirhossein Modabbernia, Vajiheh Aghamollaii, Bahador Bahrami, Joel S Winston

# Supplementary Material

# Psychiatric assessment

SF underwent a comprehensive psychiatric interview by a psychiatrist to diagnose any DSM-IV and ICD-10 psychiatric disorders. The interview was based on the Mini International Neuropsychiatric Interview for Children and Adolescents (MINI-KID) (Sheehan et al., 2010). Any positive response to MINI-KID items was subsequently evaluated in greater detail.

During the interview SF had acceptable cooperation, grooming, and affect and her neuro-vegetative functions were normal. She had a history of two interpersonal traumas three and four years ago, and history of suicidal ideation (and planning) one month and two years before the visit. She denied any suicidal acts. The reason for suicidal ideation was hopelessness due to the denial of the traumatic experience by her family. She experienced occasional nightmares related to the event, but denied avoidance of the people related to the traumatic event, alterations in arousal and reactivity, and difficulty in remembering the event. At the time of interview SF did not have suicidal thoughts or significant impairment in mood, appetite, sleep (except for occasional nightmares), and concentration. Her daily functioning was intact and her academic performance was consistently excellent. Overall SF was free of psychiatric disorders at the time.

In assessment of everyday fear sensitivity using FSSC-R, SF had a significantly lower score than controls. Indeed, in response to the question “describe how much fear you have” she selected “none” for 65 out of 80 items. She selected “a lot” for only three items (“A burglar breaking into our house”, “Getting punished by my father”, “Not being able to breathe”) and selected “some” for twelve items (“Getting punished by mother”, “Roller coaster or carnival rides”, “Bombing attacks—being invaded”, “Going to the dentist”, “Getting poor grades”, “My parents criticizing me”, “Fire—getting burned”, “Having to eat some food I don’t like”, “Failing a test”, “Being hit by a car or truck”, “Falling from high places”, “Terrorists”). Controls selected “a lot” and “some” more often to describe the fears they have [number of items (M±SD): “a lot” = 14.3±8.8; “some” = 31.5 ± 10.4]

Although SF’s response to her traumatizing life experiences could be within the normal range, it is also conceivable that the attentional bias away from threat resulted by her amygdala damage might have played a role in diminishing her emotional vulnerabilities (Pine et al., 2005). This intriguing idea is also suggested by other Urbach-Wiethe case studies (Feinstein, Adolphs, Damasio, & Tranel, 2011; Tranel, Gullickson, Koch, & Adolphs, 2006), and is directly supported by a study that showed that focal damage to amygdala is protective against post-traumatic stress disorder (Koenigs et al., 2008).

# Supplementary methods: Facial expression classification task

Face images were from Radboud Face Database (RaFD) with no additional manipulations (Langner et al., 2010). Each stimulus showed one of 6 basic expressions of emotion (‘happy’, ‘sad’, ‘fearful’, ‘angry’, ‘surprised’, and ‘disgusted’) in full color frontal headshots of 39 identities (19 females and 20 males). Stimuli were displayed on a LCD monitor (Samsung Electronics; diagonal size: 13.3 inch; resolution: 1366 × 768 pixels; refresh rate: 60 Hz) using a PC running MATLAB (the MathWorks; Natick, Mass.) with Psychtoolbox-3 (Brainard, 1997; Pelli, 1997). On each trial one image was displayed in the center of the screen (average face diagonal = 16°) and remained until the participants’ response. Face images were presented in random order. Twelve training trials (2 Identities × 6 Expressions) preceded the main task. During training, the investigator gave verbal feedback on the correctness of answers. To confirm adequate comprehension of emotional words, participants were asked to provide examples of situations that would elicit each of the emotions. All controls gave appropriate examples without difficulty. However, when probed about the word *fear*, SF stated, after a long delay, that she felt fear “… on the nights that her little brother fails to do his homework.” When asked to elaborate, her descriptions were more consistent with feelings of anxiety and anger rather than fear.

# Supplementary methods: Emotional dot-probe task

Participants sat in a light- and sound-attenuated room 40 cm from an LCD monitor (same as above). Stimulus presentation and recording of responses were controlled using Psychtoolbox-3 (Brainard, 1997; Pelli, 1997). In total each participant completed 1440 trials, yielding 160 repetitions of each Trial type × Cue duration combinations. Trials were presented in random order. Overall, the fearful face was displayed in the LVF or RVF with equal probability. To prevent fatigue a self-paced rest period was provided every 120 trials. Moreover, data collection was performed over 2 days. Each data collection session approximately took 40 minutes. Participants were instructed to fixate centrally throughout the whole experiment. They were asked to report the shape of the targets by pressing the “.” key for circles or “/” key for squares on a standard QWERTY keyboard using their right middle and index fingers. The targets stayed on the screen until the participant responded. If no response was given, the next trial would begin automatically after 2 seconds. Accuracy and speed were equally emphasized. 48 training trials (16 × 3 Trial types; Cue duration = 1000ms) were provided prior to each session in order to familiarize participants with the experimental procedure. Participants did not receive any feedback on their performance until the end of the task.

# Supplementary results: Dot-probe task performance, speed-accuracy trade-off

Participants’ performance was assessed by the frequency of making an error in reporting the shape of the target. Overall, error rate of controls were low (mean ±*SD* = 0.9% ±0.8). SF had a significantly higher error rate [3.1%; *t*(9) = 2.53; *p* = 0.032]. The overall RT of SF and controls were statistically equivalent [*t*(9) = 0.83; *p* = 0.428] and speed-accuracy trade-off was ruled out as an explanation for higher error rates of SF. Besides the specific spatial attentional effects of emotional stimuli, which is measured by the dot-probe task, task-irrelevant emotional stimuli also exert a general disruptive effect on task performance (Pereira et al., 2006). We speculated that higher errors of SF might be due to an exaggeration of the disruptive effect of the task-irrelevant emotional cues. To test this hypothesis we examined the frequency of errors in each of the trial types (see Table S1). Despite our expectation, SF had a constant frequency of errors in all three trial types. On the other hand, controls had higher errors in trials that included an emotional cue. However, the trial type × group ANOVA did not show an interaction [*F*(2, 18) = 2.89; *p* = 0.75] and only the group main effect was significant [*F*(1, 9) = 6.39; *p* = 0.032]. SF’s error rate was statistically higher than controls in the neutral condition [*t*(9) = 4.35; *p* = 0.002] but not in either congruent [*t*(9) = 1.63; *p* = 0.137] or incongruent trials [*t*(9) = 1.89; *p* = 0.091], which contained a fearful face stimulus. These results suggest that the task-irrelevant emotional stimuli caused a transient disruption of performance in controls. While SF was probably prone to higher and more sustained disruption by the emotional stimuli.

| **Table S1.** Frequency of errors in each trial type of the dot-probe task | | | | |
| --- | --- | --- | --- | --- |
| Trial type | SF |  | Controls | |
|  |  |  | *M* | *SD* |
| Incongruent | 3.1% |  | 1% | 1.1 |
| Congruent | 3.1% |  | 1.1% | 1.2 |
| Neutral | 3.1% |  | 0.5% | 0.6 |
| *M*, mean; *SD*, standard deviation. | | | | |

# Statistical considerations for single case dot-probe analysis

Multiple statistical methods have been proposed for comparing a single case with a control group. In this study we employed the modified *t* test proposed by Crawford and Howell (1998). The essence of this methodology is that the single case is treated as a sample of *N* = 1 and the control group’s mean and *SD* are treated as *sample* statistics, rather than population parameters^[[1]](#footnote-1)^. Monte-Carlo simulations show that this method outperforms alternative approaches in controlling type I error rate, especially where control numbers are low (Crawford & Garthwaite, 2006, 2012). Methods proposed by Crawford and colleagues are now the tests of choice for single-case comparisons (McIntosh & Brooks, 2011).

The logic behind Crawford & Howell’s method is readily extended to analysis of variance (ANOVA) (Corballis, 2009a, 2009b) and linear mixed modelling (LMM) (Huber, Klein, Moeller, & Willmes, 2015) procedures, thus enabling the analysis of factorial experiments where the single-case and the control subjects are tested under several conditions of the same task. The reaction time data from our dot-probe experiment had a 3-way factorial design (Group × Cue duration × Trial type).

Interactions between factors, main effects, and within-subject comparisons could all be tested using a single hierarchical design in LMM procedure. This would provide an integrated approach for analysis of our data. However, LMM is not recommended for single-subject studies with less than 15 control subjects because it provides less statistical power than the original Crawford and Howell’s modified *t* test (Huber et al., 2015). We used ANOVA for testing factorial effects and we used paired-sample *t* tests for pairwise comparison between three trial types (congruent, incongruent, neutral). These tests were applied on aggregated reaction time data (i.e., we averaged reaction times for each condition and participant separately prior to data analysis). For pairwise comparison between SF’s reaction times in each trial type, we used the Crawford and Garthwaite’s revised test for difference^[[2]](#footnote-2)^ (2005). This method is a modified paired-sample *t* test designed for testing difference between scores of a single patient on two versions of the same task. This method is well suited for detecting deficits in patient’s scores (i.e., scenarios where controls score equally well on both versions of the task but the patient is deficient in one). However, in the current study we had an instance of the opposite scenario. When cue duration was 500ms, SF showed relatively equal reaction times on incongruent and congruent trials while controls showed significantly different speeds. The validity of Crawford and Garthwaite’s method in this situation is not directly addressed in the literature. Another approach to pairwise comparison between SF’s speed in each trial type is to use unaggregated reaction times and perform conventional independent-samples *t* tests. The limitation of this approach is that its results cannot be generalized to population level because it only uses within-subject trial-by-trial variability in SF reaction times to draw statistical inferences. Therefore, its results are only valid for our single subject. To confirm the results achieved by Crawford and Garthwaite’s revised test for difference, we repeated the pairwise comparisons on SF’s data using the within-subject conventional *t* tests (see section 6 below; Confirmatory RT analyses).

# Confirmatory analyses

The responses of SF in the dot-probe task were reanalyzed using methods based on the within-subject variability of data, using linear mixed modeling procedure in SPSS. The trial-by-trial RT measurements of SF were entered into a 3 (Cue duration: 100, 500, 1000 ms) × 2 (Congruency: incongruent/congruent) ANOVA. The test revealed a significant cue duration by congruency interaction [*F*(2, 900) = 5.40; *p* = 0.005]. Main effects were not significant. To check the components of attentional biases, pairwise comparisons between RTs of each trial type at each cue duration were done using independent samples *t*-tests. Table S2 presents the results of confirmatory comparisons on SF’s RTs. These results are in agreement with results of the Crawford and Garthwaite’s revised test for difference that we have reported in the main text.

| **Table S2.** Pairwise comparisons between SF's reaction times in each trial type at each cue duration in the dot-probe task. Results are from independent samples *t-*tests based on within-subject variability in trial-by-trial measurements. | | | | | |
| --- | --- | --- | --- | --- | --- |
| Cue duration | Comparison | Difference (ms) | t(*df*) | | *p*-value |
| 100 ms | RT_incongruent_ - RT_congruent_ | 29 | 2.25 | (*300*) | 0.025 |
|  | RT_neutral_ - RT_incongruent_ | 0 | 0.00 | (*299*) | 0.998 |
|  | RT_neutral_ - RT_congruent_ | 29 | 2.22 | (*301*) | 0.027 |
| 500 ms | RT_incongruent_ - RT_congruent_ | -13 | -0.79 | (*294*) | 0.431 |
|  | RT_neutral_ - RT_incongruent_ | -37 | -2.47 | (*296*) | 0.014 |
|  | RT_neutral_ - RT_congruent_ | -50 | -3.38 | (*294*) | 0.001 |
| 1000 ms | RT_incongruent_ - RT_congruent_ | -36 | -2.62 | (*306*) | 0.009 |
|  | RT_neutral_ - RT_incongruent_ | 2 | 0.15 | (*305*) | 0.879 |
|  | RT_neutral_ - RT_congruent_ | -34 | -2.67 | (*303*) | 0.008 |
| *df*, degrees of freedom; ms, millisecond; RT, reaction time. | | | | | |

# Supplementary references

Brainard, D. H. (1997). The Psychophysics Toolbox. *Spatial Vision*, *10*(4), 433–436. http://doi.org/10.1163/156856897X00357

Corballis, M. C. (2009a). Comparing a single case with a control sample: Correction and further comment. *Neuropsychologia*, *47*(13), 2696–2697. http://doi.org/10.1016/j.neuropsychologia.2009.04.012

Corballis, M. C. (2009b). Comparing a single case with a control sample: Refinements and extensions. *Neuropsychologia*, *47*(13), 2687–2689. http://doi.org/10.1016/j.neuropsychologia.2009.04.007

Crawford, J. R., & Garthwaite, P. H. (2005). Testing for Suspected Impairments and Dissociations in Single-Case Studies in Neuropsychology: Evaluation of Alternatives Using Monte Carlo Simulations and Revised Tests for Dissociations. *Neuropsychology*, *19*(3), 318–331. http://doi.org/10.1037/0894-4105.19.3.318

Crawford, J. R., & Garthwaite, P. H. (2006). Methods of testing for a deficit in single-case studies: Evaluation of statistical power by Monte Carlo simulation. *Cognitive Neuropsychology*, *23*(6), 877–904. http://doi.org/10.1080/02643290500538372

Crawford, J. R., & Garthwaite, P. H. (2012). Single-case research in neuropsychology: A comparison of five forms of t-test for comparing a case to controls. *Cortex*, *48*(8), 1009–1016. http://doi.org/10.1016/j.cortex.2011.06.021

Crawford, J. R., & Howell, D. C. (1998). Comparing an Individual’s Test Score Against Norms Derived from Small Samples. *The Clinical Neuropsychologist (Neuropsychology, Development and Cognition: Section D)*, *12*(4), 482–486. http://doi.org/10.1076/clin.12.4.482.7241

Feinstein, J. S., Adolphs, R., Damasio, A., & Tranel, D. (2011). The human amygdala and the induction and experience of fear. *Current Biology*, *21*(1), 34–38. http://doi.org/10.1016/j.cub.2010.11.042

Huber, S., Klein, E., Moeller, K., & Willmes, K. (2015). Comparing a single case to a control group – Applying linear mixed effects models to repeated measures data. *Cortex*, *71*(December), 148–159. http://doi.org/10.1016/j.cortex.2015.06.020

Koenigs, M., Huey, E. D., Raymont, V., Cheon, B., Solomon, J., Wassermann, E. M., & Grafman, J. (2008). Focal brain damage protects against post-traumatic stress disorder in combat veterans. *Nature Neuroscience*, *11*(2), 232–7. http://doi.org/10.1038/nn2032

Langner, O., Dotsch, R., Bijlstra, G., Wigboldus, D. H. J., Hawk, S. T., & van Knippenberg, A. (2010). Presentation and validation of the Radboud Faces Database. *Cognition & Emotion*, *24*(8), 1377–1388. http://doi.org/10.1080/02699930903485076

McIntosh, R. D., & Brooks, J. L. (2011). Current tests and trends in single-case neuropsychology. *Cortex*, *47*(10), 1151–1159. http://doi.org/10.1016/j.cortex.2011.08.005

Pelli, D. G. (1997). The VideoToolbox software for visual psychophysics: transforming numbers into movies. *Spatial Vision*, *10*(4), 437–442. http://doi.org/10.1163/156856897X00366

Pereira, M. G., Volchan, E., de Souza, G. G. L., Oliveira, L., Campagnoli, R. R., Pinheiro, W. M., & Pessoa, L. (2006). Sustained and transient modulation of performance induced by emotional picture viewing. *Emotion (Washington, D.C.)*, *6*(4), 622–34. http://doi.org/10.1037/1528-3542.6.4.622

Pine, D. S., Mogg, K., Bradley, B. P., Montgomery, L., Monk, C. S., McClure, E., … Kaufman, J. (2005). Attention bias to threat in maltreated children: Implications for vulnerability to stress-related psychopathology. *American Journal of Psychiatry*, *162*(2), 291–296. http://doi.org/10.1176/appi.ajp.162.2.291

Sheehan, D. V, Sheehan, K. H., Shytle, R. D., Janavs, J., Bannon, Y., Rogers, J. E., … Wilkinson, B. (2010). Reliability and validity of the Mini International Neuropsychiatric Interview for Children and Adolescents (MINI-KID). *J Clin Psychiatry*, *71*(3), 313–326. http://doi.org/10.4088/JCP.09m05305whi

Tranel, D., Gullickson, G., Koch, M., & Adolphs, R. (2006). Altered experience of emotion following bilateral amygdala damage. *Cognitive Neuropsychiatry*, *11*(3), 219–232. http://doi.org/10.1080/13546800444000281

1. The modified independent-samples *t* test to compare a single patient with a small control group based on the Crawford and Howell’s method:

$$t_{n-1}= \frac{X^{*}- \bar{X}}{s\sqrt{(n+1)/n}}$$

   Where $X^{*}$ is the patient’s score, $\bar{X}$ and $s$ are the mean and *SD* of scores in the control sample, and $n$ is the size of the control sample. [↑](#footnote-ref-1)
2. The modified paired-samples *t* test to check the difference between a single patient’s scores in two versions of the same task:

$$t_{n-1}= \frac{X^{*}-Y^{*}}{\sqrt{(s_{X}^{2}+ s_{Y}^{2}-2s_{X}s_{Y}r_{XY})}}$$

   Where $X^{*}$ and $Y^{*}$ are the patient’s scores in two versions of the task, $s_{X}$ and $s_{Y}$ are the *SD* of the controls’ scores, $r_{XY}$ is the correlation between the scores of controls, and $n$ is the size of the control sample. The term under the radical sign is the variance of the difference between two task versions for controls. [↑](#footnote-ref-2)
