# Supplementary material for: Attentional bias towards and away from fearful faces is modulated by developmental amygdala damage
Source: Cortex. 2016 Aug;81:24–34. doi: 10.1016/j.cortex.2016.04.012 (PMC4962776; doi:10.1016/j.cortex.2016.04.012)
Supplement: Supplementary file 2 [file mmc2.docx]

| **Table 1.** Mean reaction times (in ms) on congruent, incongruent, and neutral trials for SF and controls at each cue exposure duration. | | | | | |
| --- | --- | --- | --- | --- | --- |
|  |  | SF |  | Controls *(n = 10)* | |
| Cue duration | Trial type | *M* |  | *M* | *SD* |
| **100 ms** | Incongruent | 667 |  | 585 | 50 |
|  | Congruent | 638 |  | 593 | 50 |
|  | Neutral | 667 |  | 584 | 46 |
| **500 ms** | Incongruent | 657 |  | 579 | 40 |
|  | Congruent | 670 |  | 563 | 37 |
|  | Neutral | 620 |  | 570 | 33 |
| **1000 ms** | Incongruent | 623 |  | 585 | 63 |
|  | Congruent | 659 |  | 568 | 63 |
|  | Neutral | 625 |  | 564 | 62 |
| *M*, mean; ms, millisecond; RT, reaction time; S*D*, standard deviation. | | | | | |
